# Advancing the Use of High-Performance Graphene-Based Multimodal Polymer Nanocomposite at Scale

**DOI:** 10.3390/nano8110947

**Published:** 2018-11-17

**Authors:** Ibrahim A. Ahmad, Krzysztof K. K. Koziol, Suleyman Deveci, Hyun-Kyung Kim, Ramachandran Vasant Kumar

**Affiliations:** 1 Department of Materials Science and Metallurgy, University of Cambridge, 27 Charles Babbage Rd, Cambridge CB3 0FS, UK; iaiaa2@cam.ac.uk; 2 Enhanced Composites and Structures Centre, School of Aerospace, Transport and Manufacturing, Cranfield University, Cranfield MK43 0AL, UK; k.koziol@cranfield.ac.uk; 3 Innovation Centre, Borouge Pte. Ltd., PO Box 6951, Abu Dhabi, UAE; suleyman.deveci@borouge.com; 4 Gwangju Bio/Energy R&D Center, Korea Institute of Energy Research (KIER), 270-25 Samso-ro, Buk-gu, Gwangju 61003, Korea

**Keywords:** graphene, multimodal-high density polyethylene, melt extrusion, polymer, nanocomposite, polymer degradation, dispersion and distribution of graphene

## Abstract

The production of an innovative, high-performance graphene-based polymer nanocomposite using cost-effective techniques was pursued in this study. Well-dispersed and uniformly distributed graphene platelets within a polymer matrix, with strong interfacial bonding between the platelets and the matrix, provided an optimal nanocomposite system for industrial interest. This study reports on the reinforcement of high molecular weight multimodal-high-density polyethylene reinforced by a microwave-induced plasma graphene, using melt intercalation. The tailored process included designing a suitable screw configuration, paired with coordinating extruder conditions and blending techniques. This enabled the polymer to sufficiently degrade, predominantly through thermomechanical-degradation, as well as thermo-oxidative degradation, which subsequently created a suitable medium for the graphene sheets to disperse readily and distribute evenly within the polymer matrix. Different microscopy techniques were employed to prove the effectiveness. This was then qualitatively assessed by Raman spectroscopy, X-ray diffraction, rheology, mechanical testing, density measurements, thermal expansion, and thermogravimetric analysis, confirming both the originality as well as the effectiveness of the process.

## 1. Introduction

Multimodal high-density polyethylene (HDPE) is an engineered thermoplastic semi-crystalline polymer, which is widely used in automotive, films, pressure pipes and fittings, bottles, tubes, and cables jacketing [1,2,3,4,5]. It is a hybrid of at least two distinct polyethylene components, wherein each constituent has a different density and different molecular weight fractions [1,2,3,4,5]. This allows flexibility in engineering its microstructure to meet the desired balance of properties for concrete practical applications. Nevertheless, multimodal HDPE can be further improved, for example, with the addition of fillers or reinforcements, in order to overcome deficiencies in their mechanical or thermal properties [5,6,7,8,9,10,11,12,13,14,15,16,17,18]. It is feasible that a substantial benefit could be attained by strengthening the multimodal-hydrophobic polymers with graphene (g), deriving new and unique nanocomposite properties [5,6,7,8,9,10,11,12,13,14,15,16,17,18,19,20,21,22]. However, a proper dispersion and distribution of graphene platelets within the nonpolar polyolefin matrix is still a major challenge [6,15,16,17,18,19,20,21,22]. Irreversible agglomerates are formed through the van der Waals forces between the 2D-platelets, as the large surface area of the graphene platelets leads to the creation of interfacial regions, causing them to spontaneously restack themselves [4,6,15,16,17,18,19,20,21]. This creates defects which behave as voids, introducing degradation into the polymer matrices. Though numerous methods for producing polymer-graphene nanocomposites have recently emerged, each method is limited by its compatibility with only certain types of graphene and polymers, requiring chemical modifications on both constituents of the nanocomposite [6,15,16,17,18,19,20,21]. The fabrication techniques can also alter the pristineness of graphene by introducing structural defects on the graphene basal plane [15,16,17,18,19,20,21,22]. In addition, the production utilizes large amounts of solvent and supplementary chemicals, which incurs higher costs, as well as raising environmental, health, and safety concerns [15,16,17,18,19,20,21,22].

**Figure 1:**
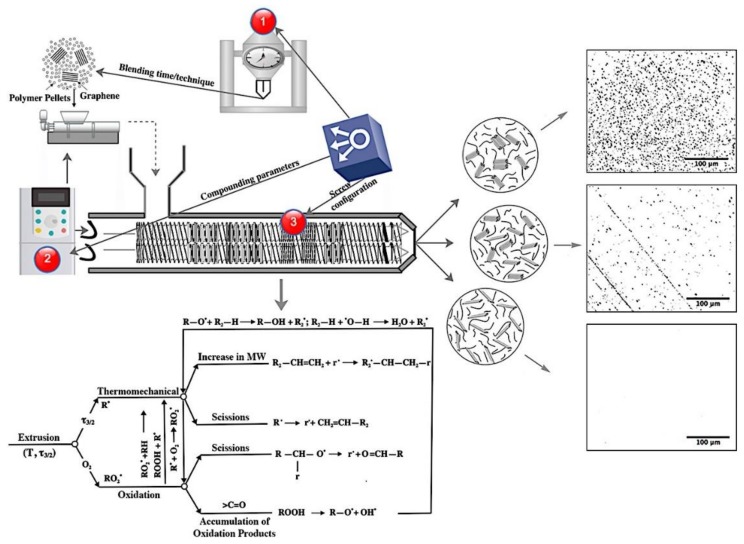
Simple schematic representation of the method followed in the present study. Gol’dberg–Zaikov model represents the general reaction mechanisms of all the thermo-mechanical and thermo-oxidative degradations that can occur during the melt extrusion of a polyethylene [24,25]. R represents the side chain of any hydrocarbon functional groups, r is the very short side chain of any hydrocarbon functional groups, MW is the molecular weight, ˙ denotes an active free radical site, and *τ_3/2_* is the shear stress in x and y directions.

It is therefore the topic of this study to introduce a more cost-effective, optimal way of fabricating a nanocomposite of high molecular weight multimodal-HDPE matrix, reinforced with a bottom-up graphene. These polymers are indeed widely used in a long-term application in an extreme environment, which includes hydrostatic, thermal, and environmental stresses [5,21,23]. Herein we report a novel method for the preparation of high-performance polymer-graphene nanocomposite (PE-g) via melt intercalation, using a co-rotating intermeshing twin-screw extruder. Depicted in Figure 1 is a simple schematic diagram of the fabrication method followed in the present study.

In the present work, we attempted to degrade the polymer to a sufficient level, through thermo-oxidative, as well as thermo-mechanical degradation during the melt extrusion process. This created a compatible medium for the graphene to disperse and distribute thoroughly within the polymer matrix. The polymer is consequently able to interact physically or chemically with the residual oxygen functional groups at the graphene surface which contains almost 5% oxygens, or through the short molecules introduced by thermo-mechanical degradation, with defective sp^3^ functional group on either the surface, or at the edge of the graphene sheets. Accordingly, a better stress transfer can potentially be achieved through the strong interfacial bonding created between graphene platelets and the polymer matrix. Achieving a thorough dispersion and distribution of graphene within the multimodal-HDPE, by melt intercalation, via co-rotating intermeshing twin-screw extruder, has never yet been reported according to the authors’ knowledge. The results of this research provide greater insight into different melt intercalation factors, affecting the multimodal HDPE-graphene nanocomposite performance and criterion for effectively producing the next generation of black multimodal-polyethylene compounds for use in high-pressure pipes, automotive, and energy cable applications [5,6,7,8,9,10,11,12,13,14,15,16,17,18,19,20,21,23]. 

## 2. Experimental

### 2.1. Materials

Unstabilized high-density polyethylene powders, produced with Ziegler Natta catalyst via proprietary Borstar process, (Borouge, United Arab Emirates), with a melt flow rate of about 7.5 g/10 min (190 °C, 21.6 kg), Mw = 280 kg/mole, Mn = 8.49 kg/mole, Mw/Mn = 33, and a density of 950 kg/m^3^ were used in this study. The multimodal high-density polyethylene matrix used in the present study was engineered specifically for nanocomposite applications. According to the production process technology, the melt flow rate (MFR) of the polymer gives an indication that the split MFR ratio between the reactors is significantly high [1,2,3,4]. The antioxidants’ masterbatch containing Irganox 1010 and Irgafos 168 were added to the polymers at 0.5 wt.% for optimum stabilization during processing. Graphene was supplied by the FGV Cambridge Nanosystems Ltd. (Cambridge, United Kingdom), with ≥95% carbon purity, bulk density of 0.0266 g/ml, thickness < 1.0 nm, and flake size range of 150–500 nm. Carbon Black powder was provided by Orion Engineered Carbons GmbH (Frankfurt am Main, Germany), with ≥92 cc/100g oil absorption number, ash content of 0.10%, sulphur content of 0.10%, tint strength of 103%, average primary particle size of 20 nm, and a density of 1.7-1.9 g/cm^3^ at 20 °C. 

### 2.2. Nanocomposite Preparation

**Figure 2:**
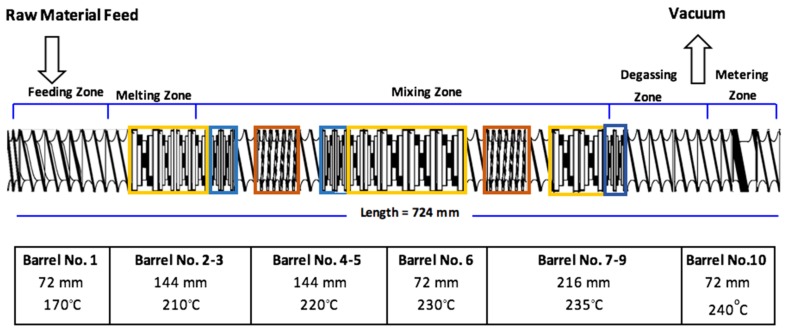
Modular extrusion screw configuration based upon individual barrel sections and screw elements. The color boxes show the position of the dispersive and distributive kneading elements along the screw shaft (length of 724 mm).

The graphene-based multimodal-HDPE nanocomposites (PE-g) were prepared via melt intercalation using a Coperion ZSK 18 twin extruder, with a screw diameter of 18 mm and a barrel length of 720 mm (L/D = 40). The screw rotation speed (rpm) was 600 min-1, barrel temperature profile was in the range of 170-240 °C (see Figure 2), and feed rate was between 1-2 kg/hr. Both the graphene and dry polyethylene powders were fed separately into the extruder via a spiral flow screw Brabender ISC-CM plus feeder. The nanofillers were fed at 0.1, 0.5, 1, 2, and 5 wt.% loadings. In order to prevent the polymer from severe degradation, an antioxidant masterbatch was simultaneously added through a side feeder, with the total loading of 0.5 wt.%. The extruded pellets were subsequently compression molded to about 0.4 mm thickness, following ISO 293 under 5 MPa, at a temperature of 200 ºC. This was undertaken via a compression molding platen press (Dr. Collin P 400 M, Ebersberg, Germany), for an overall programming cycle of 32 min, at a heating and cooling rate of 15 ºC/min. The specimens were successively conditioned at 23 ± 2 °C and 50 ± 5%, for at least 48 h, prior to being tested.

A schematic of a modular twin-extrusion screw configuration used in the present study is given in Figure 2. There are four main types of screw elements generally used in co-rotating twin screw extruders; forward and back flow convening elements (unboxed), kneading elements for dispersive mixing purposes (yellow and blue boxes), and toothed mixing elements for distributive mixing purposes (orange box) [26,27,28,29,30]. The screw consisted of 30% of 2-flighted right-handed normal and wide kneading elements (yellow box), with a 45º staggering angle, 9% of 16-flighted right-handed mixing elements (orange box), 6.7% of the 2-flighted left-handed narrow kneading elements (blue box) distributed over each dispersive segment. These percentages were based on the ratio of the mixing elements length to the total length of the screw shaft (720 mm).

Since graphene has the ability to shield the polymer from heating and becoming completely molten by enhancing the thermal stability in the feeding and melting zones [17,29,30,31,32,33], the nanocomposite constituents were simultaneously fed from separate feeders into the extruder to prevent graphene platelets from stabilizing around the multimodal-HDPE powders. A long dispersive segment was incorporated in the melting zone to increase the fusion rate of the polymer prior to entering the homogenization zone. The left-handed narrow kneading elements (blue boxes) were placed on each dispersive segment to melt the polymer entirely in the melting zone, and increase the residence time at each dispersive segment. It induces a distribution mixing rather than dispersive (shearing) mixing, especially as its pitch length is very narrow [26,27]. The two distributive elements (orange boxes) were placed between long dispersive segments in order to keep the nanocomposite constituents under continuous high-pressure, and to cause the dispersed (sheared) graphene sheets to instantaneously be pushed away. One of the distributive elements (orange box) was placed between left-handed narrow kneading elements to increase the residence time in a narrower axial length, at the beginning of the homogenization zone, by generating a reverse flow with the use of advancing discs which tend to compress the fluid. This modular assembly build enabled the polymer to degrade to a sufficient level in the targeted zone, under combined elongation and shear forces, prior to entering the homogenization zone. Resultantly, melting the polymer could be completed at the first kneading segment in the melting zones, preventing the graphene platelets from moving smoothly and re-connecting together through the van der Waals’s interactions. 

### 2.3. Characterization

#### 2.3.1. Polarized Light Microscope (PLM)

Optical microscopy analyses were conducted on ZIESS Axio scope.A1 HAL 100/HBO 100, operated with an AxioCam MRc 5 camera, and AxioVision software. Film samples were sectioned to a thickness of 15 μm, using a fully automated rotary microtome Leica RM2265 (Leica microsystems, Wetzlar, Germany). 

#### 2.3.2. Transmission Electron Microscope (TEM)

Transmission electron microscopy (TEM) was performed using a Hitachi HT7700, at an accelerating voltage of 120 kV. Film samples were cryo-sectioned to a thickness of ~80 nm at -125 °C, using a Leica EM UC7/FC7 Cryo-Ultra-microtome. 

#### 2.3.3. Density Measurement

Density measurement was performed with an analytical balance, equipped with a density measurement kit (Metter Toledo XP205, Zurich, Switzerland), following an ASTM D792-method B, based on the Archimedes’ principle where the weight of the sample immersed in an n-dodecane fluid decreases by an amount equal to the displacement of the liquid weight. 

#### 2.3.4. Raman Spectroscopy

Raman measurements were carried out using Renishaw inVia confocal Raman microscope with 633 nm and 532 nm lasers. With the exception of the deformation test, all the Raman data was collected using He-Ne ion laser with a wavelength of 633 nm (red, 1.96 eV). However, an Nd-YAG laser with a wavelength of 532 nm (green, 2.33 eV) was used to evaluate the stress-transfer along the interfacial surface between the polymer matrix and reinforcement. The dog-bone specimens, having been prepared for tensile testing, were deformed in a three-point bending rig. The strain (ε_f_) was measured by calculating the deflection of the beam at the mid-span (δ), following the equation ε_f_=6 δ t/L^2^, where *t* is the thickness of the beam specimen and *L* is the span between the supports [6].

#### 2.3.5. X-ray Diffraction (XRD)

X-ray diffraction (XRD) measurements were performed using a Bruker D8-Advance diffractometer equipped with Cu Kα1 radiation (λ = 1.54060 Å). The diffraction patterns were recorded with a step size of 0.15087°, and dwell time of 5s. 

#### 2.3.6. Scanning Electron Microscope (SEM)

A scanning electron microscope (SEM) was utilized to assess the cryofractured cross-section surface of the uncoated polymers, using an FEI QUANT 250 FEG SEM, at an accelerating voltage of 2 kV. Samples were notched prior to being submerged in liquid nitrogen for ten minutes. 

#### 2.3.7. Tensile Testing

Tensile properties of the nanocomposites such as tensile modulus, stress and strain at both yield and break, as well as other aspects of the tensile stress-strain curve were studied on the stamped ISO 527-2 dog-bone specimens, type 1B. This was achieved using die punch equipment (Elastocon EP 02, Sweden) on the compression molded samples. These were measured with the Zwick/Roell Universal Testing Machine (UTM)–Z050, using a load cell of 2.5 kN, grip-to-grip separation of 115 mm, a gauge length of 50 mm, and a cross-head speed of 50 mm/min. A contact type extensometer (Zwick MultiXtense, Germany) was used to measure the strain of the specimen. The results were based on a minimum of 6 specimens. 

#### 2.3.8. Thermomechanical Analyzer (TMA)

Q400 Thermomechanical analyzer (TMA) was employed to determine the bending properties of the nanocomposites with dynamic mode through the 3-point bending test. Samples were cooled to -150 °C at the cooling rate of 3 °C/min for 10 min, and subsequently heated to 150 °C at the heating rate of 3 °C/min. The modulate force was 0.01 N at a frequency of 1 H_z_ in an ambient gas atmosphere (50 mL/min), using a wedge-shaped quartz probe. The dimension of the samples was 10 × 3.4 × 0.5 mm^3^. The same equipment was employed for studying the coefficient of thermal expansion (CTE) via a flat-tipped standard expansion probe in an ambient gas atmosphere (50 mL/min). Flat samples with dimensions of 6 × 6 × 4 mm^3^ were heated at 3 °C/min from room temperature to 100 °C. They were then held for 10 min, then cooled to 0 °C at the same rates of 3 °C/min, then held for 10 min, and subsequently heated at 3 °C/min to 120 °C, under a constant load of 0.05 N. The displacement was reset to zero at the start of the last sequence where the measurement started. 

#### 2.3.9. Rheology Analysis

Simple qualitative characterization related to an indirect measurement of molecular weight and processability of the polymer was studied upon the melt-flow rate (MFR). Pellets of 3-8 g were charged into a cylinder at 190 °C under the load of 21.6 kg, which was achieved using the Melt Indexer MI-4 manufactured by GÖETTFERT Werkstoff–Prüfmaschinen GmbH (Buchen, Germany), following ISO 1133 procedure B. The rheological behavior of the samples was studied using stress-controlled rotational rheometer, an Anton Paar Physica MCR 301 with CTD450 heating unit, at 190 °C under a nitrogen atmosphere. The compression molded sample, weighing 1.5 g, 25 mm in diameter, and 1.5 mm thick was conditioned at 40 °C for 48 h. The sample was then placed onto a 25 mm parallel plate fixture and trimmed to a thickness of 1.2 mm by slowly lowering the upper plate. Dynamic frequency sweep was conducted from 500 to 0.0154 rad/s at 5% strain. The reason for starting from the maximum frequency was to avoid sample degradation under high temperature and low angular speed. The polydispersity index (PDI) was measured as follows [33]:

(1) $$PDI=\frac{100\;000}{G^{\prime }\left(\omega_{COP}\right)},\;\;\omega_{COP}=\omega \left(G^{\prime }=G^{\prime \prime }\right)$$

where *G’* is the storage shear modulus, *G”* is the loss shear modulus, *ω* is the angular frequency, and $\omega_{COP}$ is the crossover frequency obtained from the intersection of storage modulus and loss modulus in a log-log scale of a frequency sweep test. 

#### 2.3.10. Thermogravimetric Analysis (TGA)

Thermogravimetric analysis (TGA) was carried out with Q500 TGA (TA instruments, New Castle, USA) with a heating rate of 10 °C /min from room temperature to 1000 °C in a nitrogen atmosphere. High-resolution (Hi-Res)-dynamic mode was performed with a sensitivity of 2.00 and a resolution of 4 °C. 

## 3. Results and Discussion

A twin-screw extrusion system with a modular screw configuration was utilized in this study to fabricate a polymer nanocomposite with well-dispersed and uniformly distributed graphene flakes. The screw configuration was optimized after several trials, starting with a screw design employed for compounding such polymers with a high-volume fraction of nanoparticles (carbon black) or nanoclays (talc). A combination of different elements was utilized and arranged according to mixing requirements and material properties to be attained [26,27,28,29,30,31,32]. A schematic of the optimal screw configuration for the studied nanocomposite model is given in Figure 2. 

### 3.1. Morphology of Graphene Sheets in a Nanocomposite Matrix

**Figure 3:**
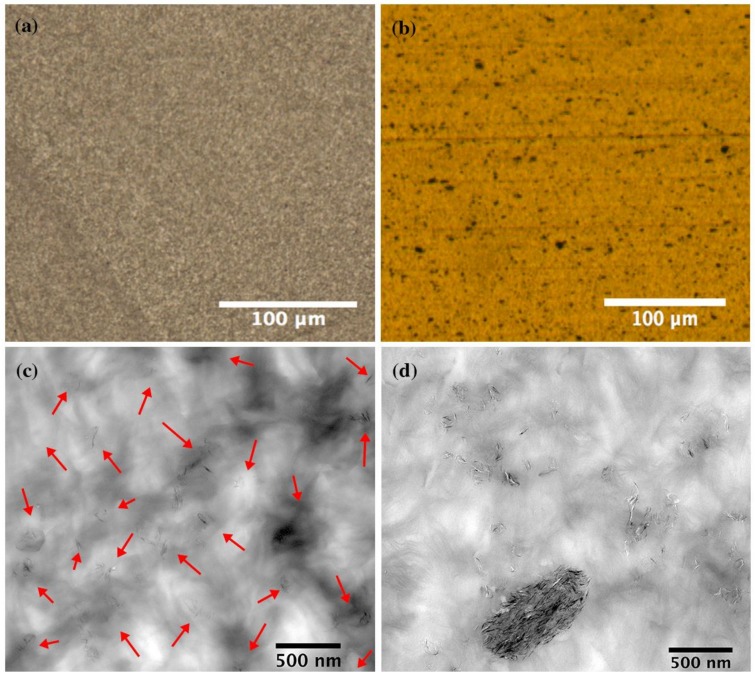
(**a**,**b**) Light microscopy images and (**c**,**d**) TEM images show the dispersion and distribution of 1 wt.% loading of graphene platelets within the multimodal-HDPE matrix (PE-g-1%). Images for the similar nanocomposite produced by a pre-existing processing protocol (**right**), were compared with PE-g-1% produced in this study (**left**). The TEM and light microscopy images were taken at 10k and 20x, respectively.

The tailored process included designing a suitable screw configuration, paired with coordinating extruder conditions and blending techniques. This subsequently created a suitable medium for the graphene platelets to disperse readily, and distribute thoroughly within the multimodal-HDPE matrix, as demonstrated in Figure 3a,c. The mean particle size of the detected graphene particles and %area fraction (200 × 200 µm^2^) was around 0.5 µm^2^ and 0.0063, respectively. Graphene monolayers are transparent under an optical microscope, opacity of 2.3 ± 0.1%, while the optical loss become greater in the wrinkled and overlapped samples [34,35]. L. J. Cote et al. [35] found that the average light scattering from the wrinkled region was about 3.7 times that of the overlapped areas. For the nanocomposite produced using the pre-existing commercial approach, however, the mean particle size of the graphene agglomerates was calculated to be 4.12 µm^2^, with maximum particle size of around 4.7 µm^2^, and a %area fraction of 79.4 (see Figure 3b,d). The %area fraction and mean particle size were calculated based on transmission electron microscope (TEM) and light microscopy analysis, graphene particles of less than 0.05 µm^2^ or 500 nm were excluded from the calculations, i.e. the average lateral size of graphene platelets ranges between 150–500 nm. A decrease in the %area fraction means a better distribution and fewer agglomerates. 

### 3.2. Dispersion and Distribution of Graphene Platelets Within the Nananocomposite Matrix

**Figure 4:**
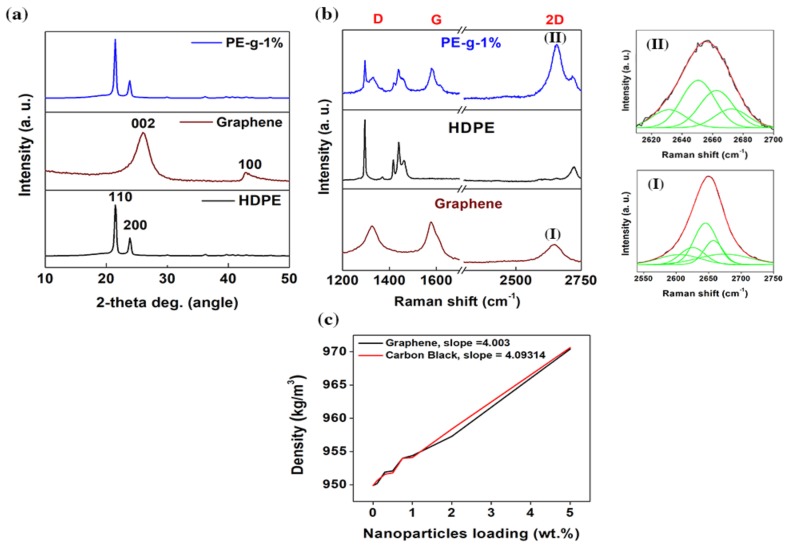
Dispersion and distribution of graphene platelets within the polymer matrix. (**a**) XRD patterns of the neat multimodal-HDPE, graphene powder, and PE-g-1%. (**b**) Overlaid Raman spectrum of the neat multimodal-HDPE, graphene powder, and PE-g-1%. The measured 2D Raman bands with 1.96 eV laser energy of graphene (**I**) and PE-g-1% (**II**) are fitted with four and five Lorentzians, respectively. (**c**) Density measurement of graphene/multimodal-HDPE (PE-g), and carbon black/multimodal-HDPE (PE-CB) nanocomposites as a function of nanofiller loading (0.1, 0.5, 1, 2, and 5 wt.%).

Figure 4a shows the X-ray diffraction (XRD) patterns of the neat multimodal-HDPE, graphene powder, and PE-g-1% samples. The diffraction peak (002) appeared in the XRD pattern of graphene at 2θ = 26.07° and exhibited a broad band with a corresponding d-spacing of 0.3414 nm (Bragg’s law), and average thickness of 1.941 nm (Scherrer’s equation), whereas the weak diffraction peak (100) was observed at 2θ = 42.89° [36,37]. This indicates the sample flakes consisted of 5-6 graphene layers, which is consistent with the TEM images shown in Figure S4. The weak intensity of these two characteristic diffraction peaks is due to the 2D nature of graphene, especially those with very few layers [38]. Interestingly, the XRD pattern of the PE-g-1% is similar to that of a neat multimodal-HDPE matrix, only showing the crystalline diffraction peaks {(110) and 200)} of the neat multimodal-HDPE matrix. Clearly the XRD results demonstrated that the graphene platelets almost exfoliated into individual sheets and dispersed well in the polymer matrix after the extrusion processing [36,37,38,39,40,41,42]. 

Overlay Raman spectra of the neat multimodal-HDPE, graphene powder, and PE-g-1% samples, are shown in Figure 4b. The three intense peaks appeared in the Raman spectra of graphene at 1327 cm^−1^, 1577.5 cm^−1^, and 2646 cm^−1^, representing the characteristic D-band, G-band, and 2D-band peaks, respectively [43,44,45]. The G-band arises from the bond stretching of the sp^2^ carbon atoms (chains or rings), while the breathing modes of the sp^2^ carbon atoms in a hexagon ring gives rise to the D-band [43,44,45]. The D-band, therefore, requires a defect to be activated (by disorder or at the edge) [43,44,45]. It originates from one iTO phonon mode around the *K* point by double resonance, whereas the overtone of the D’ and D-bands gives rise to 2D’ and 2D-bands [43,44,45]. The 2D (or 2D’) peak does not require a defect for its activation, because it originates from the two iTO phonons with opposite momentum near Brillouin zone [43,44,45]. The position, full width at high maximum (FWHM), intensity ratio (*I*_2D_/*I*_G_), and Lorentzian fittings of the 2D peak provide good correlation with the number of layers of graphene in a flake sample [43,44,45,46,47]. In Figure 4bI, the 2D-band of graphene sample is fitted by five Lorentzians, with an overall FWHM of 65.52 cm^−1^. Z. Lin et al. [46] and E. Dervishi et al. [47] in fact used up to five Lorentzian peaks to fit the 2D-band of their few-layer graphene produced by a bottom up approach. For PE-g-1%, the three prominent characteristic D, G, and 2D peaks associated with graphene were observed at 1327 cm^−1^, 1582.5 cm^−1^, and 2658 cm^−1^, respectively. The 2D-band of the nanocomposite shown in Figure 4bII, is red-shifted from 2646 cm^−1^ to 2658 cm^−1^, fitted by four Lorentzians, with an overall FWHM decreased from 65.52 cm^−1^ to ~53 cm^−1^. Four fitted Lorentzians, each with a FWHM of ~ 24 cm^−1^, most likely arose from the asymmetry between the valence and conduction bands present in the bilayer graphene [43,44,45,46,47]. Besides, the increase of the (*I*_2D_/*I*_G_) from 0.98 for graphene to 1.55 for PE-g-1%, reveals the reduction of the graphene layers [43]. Overall, these results indicate that the graphene platelets are indeed dispersed (thinned) through the melt extrusion process. 

Figure 4c depicts the density (*ρ*_c_) of the graphene/multimodal-HDPE (PE-g) and carbon black/multimodal-HDPE (PE-CB) nanocomposites at nanofiller loadings of 0.1, 0.5, 1, 2, and 5 wt.%. The PE-CB sample is a commercial grade, produced based on the same polymer matrix, but reinforced by a carbon black with a density of 1700-1900 kg·m^−3^. A monolayer graphene is made up of covalently-bonded sp^2^-hybridised carbon atoms, densely packed in a honeycomb lattice [21,22]. Therefore, the density of a defect-free monolayer graphene, with a thickness of 0.142 nm, is estimated to be around 2175 kg·m^−3^ [48]. On the other hand, carbon black is composed of primary particles that are permanently fused together through the covalent bonds, into an aggregate structure [49]. Each primary particle is made up of imperfect crystallites of turbostratic graphite structure, which are twisted into each other throughout the aggregates [49]. Accordingly, the graphene used in this study is likely to have a density closer to the carbon black density than a monolayer graphene, i.e., defective surface structure through the oxygen-containing functional groups (see Supplementary Materials Figure S3). As is evident from Figure 4c, the density of the multimodal-HDPE matrix (*ρ*_m_ = 950 kg·m^−3^) increased linearly with the addition of the nanofillers, i.e., densities of both nanocomposites increased by the same amount. The slope values are calculated at 4.003 for PE-g and 4.093 for PE-CB, suggesting that the graphene platelets were homogenously dispersed and distributed throughout the polymer matrix. An increase in the nanocomposite density is attributed to the high density of the reinforcements (*ρ*_r_) employed to reinforce the polymer matrix, according to the equation of the form (*ρ*_c_ = 1/(*W*_r_/*ρ*_r_) + (*W*_m_/*ρ*_m_)), where *W*_r_ and *W*_m_ are the weight fractions of reinforcement and matrix, respectively [4]. With a greater incorporation of high-density reinforcement, a higher nanocomposite density is obtained. In the case of agglomeration however, most of the graphene platelets will be lost in the accumulation, thereby the increase in the nanocomposite density remains relatively small.

### 3.3. Interfacial Adhesion Strength between Graphene and Polymer Matrix

**Figure 5:**
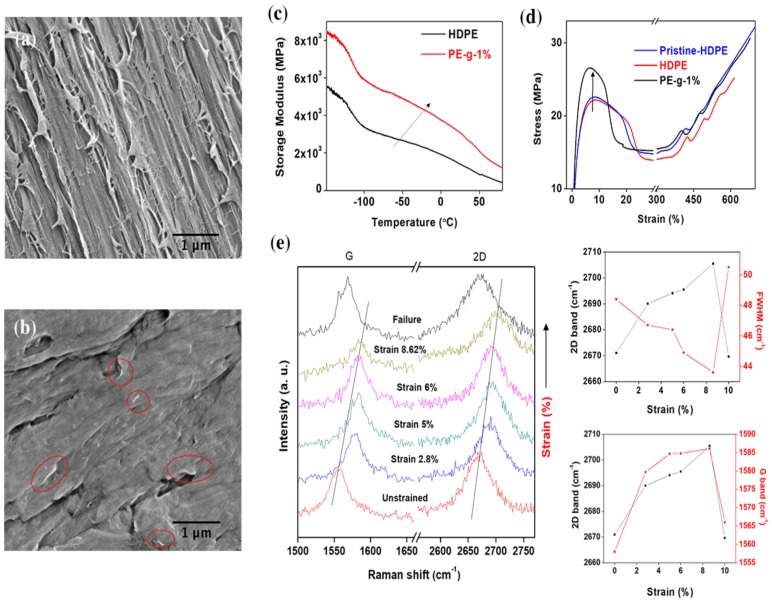
Assessment of the interfacial adhesion strength between graphene sheets and polymer matrix. SEM images of a cross-section fracture surface from (**a**) neat multimodal-HDPE and (**b**) PE-g-1%. (**c**) Dynamic-thermomechanical analysis (DTMA) of the neat multimodal-HDPE and PE-g-1%. (**d**) Tensile stress-strain curves for the pristine multimodal-HDPE (non-extruded), neat multimodal-HDPE (extruded), and PE-g-1%. The pristine polymer is the powder polyethylene. (**e**) Shift with strain of the 2D and G Raman bands of the graphene during deformation upon PE-g-1% nanocomposite (laser excitation energy 2.33 eV). The corresponding 2D and G Raman shifts as a function of applied strain are shown in the two graphs on the right.

The interfacial adhesion strength between graphene sheets and a polymer matrix can be explored through the microscopic examination of cryofractured cross-sectional surfaces. Shown in Figure 5a is a SEM image of the neat multimodal-HDPE surface exposed by cryofracture. The SEM micrograph exhibits fibrils with various extents of surface fibrillation in the draw direction. The occurrence of fibrils may suggest that the fracture was due to chain slippage or scission in crystalline (long fibrils) and amorphous (short fibrils) regions [50]. Contrastingly, graphene was shown to have a significant effect on the microstructure of the adjacent polymer as evident by changes to the fibrous morphology of the PE-g-1% shown in Figure 5b. The SEM micrograph of the nanocomposite exhibits a number of graphene platelets protruding out of the fracture surface of the polymer matrix, i.e. embedded and strongly tied to the matrix.

These flakes are well dispersed and evenly distributed within the multimodal-HDPE matrix, which may have formed a continually interconnected network structure throughout the matrix [50,51,52,53,54]. Interestingly, the fractured surface of the nanocomposite become rough, compared to that of the unfilled multimodal-HDPE. Conceptually, the fracture toughness is quantified by the amount of the energy absorbed per unit crack extension [52]. Therefore, the significant change in the breaking (crack propagation) mechanism accordingly suggests that the strong interfacial bonding between the polymer matrix and graphene platelets likely split the material into cavities and molecular bundles under large loading [50]. The facilitated stress transfer along the large interfacial area between the reinforcement and matrix is expected to potentially display mechanical reinforcement [50,51,52,53]. In Figure 5c, the storage modulus measured by dynamic thermomechanical analysis (DTMA) increased by 75%, 84%, and 118% at -100 °C, -50 °C, and 23.5 °C, respectively. The tensile modulus increased by ≥35%, from 835 ± 13 MPa for neat multimodal-HDPE, to 1135 ± 17 MPa for PE-g-1%, as shown in Figure 5d. Moreover, the maximum tensile strain increased by 11%, from 615 ± 43 % for neat multimodal (extruded), to 680 ± 31% for the PE-g-1%. This increase in the tensile strain was possibly preceded by a prolonged exposure of the neat polymer to a high temperature in the extruder, under a combined high shear and elongation forces [55]. Thus, graphene has most likely acted as an antioxidant and protected the polymer from excessive thermo-oxidative degradation [55,56]. The maximum tensile strain of the nanocomposite is therefore compared to a non-extruded multimodal-HDPE for verification. Interestingly, the tensile strain decreased from >800% for neat multimodal-HDPE (non-extruded) to only 680 ± 31% for the PE-g-1%. This latter subject will be discussed in greater detail later in this study. Nevertheless, this indicates that graphene reinforced the polymer through the heat transfer from the polymer matrix to graphene platelets along the interface.

The interfacial adhesion strength between the polymer matrix and graphene platelets was further investigated by the stress-induced Raman band shifts [57,58,59,60]. In Figure 5e, the 2D and G Raman bands of graphene in a nanocomposite shifted to higher wavenumbers as a function of applied strain, suggesting that the graphene platelets went into biaxial compression as reported in the literature [57,58]. Beyond ~9% strain however, these two bands reverted closer to that of the unstrained peak positions, due to relaxation of the graphene sheets upon debonding between the nanocomposite constituents [57,58,59,60]. The 2D and G Raman bands have significantly downshifted after ~9% strain, by ~34 cm^−1^ and 28 cm^−1^, respectively. Surprisingly, the 9% strain is around the yield point as can be seen in the stress-strain curve shown in Figure 5d. Overall, the results show that a strong interfacial bonding is created between graphene sheets and polymer matrix [57,58,59,60].

### 3.4. Rheology and Thermal Stability Performance of a Nanocomposite

**Figure 6:**
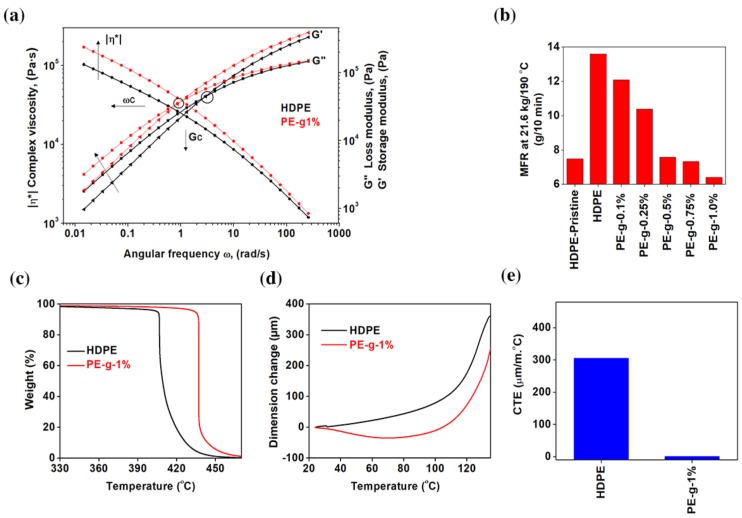
Thermal stability performance and rheological behaviors. (**a**) Dynamic frequency sweep measurements performed at 190 °C. *ω_C_* is the crossover frequency point and *G_C_* is the crossover modulus point in a log–log scale. (**b**) Melt flow rate (MFR) measurements of PE-g nanocomposites as a function of graphene loading (0.1, 0.25, 0.5, 0.75, and 1.0 wt.%). (**c**) Thermogravimetric thermograms performed in N_2_ atmosphere. (**d**) Dimensional change as a function of temperature. (**e**) Coefficient of thermal expansion (CTE) measured at temperature difference range of 30-105 °C.

Figure 6a-b shows the rheological behaviour of neat multimodal-HDPE and PE-g-1%. Shown in Figure 6a is the pseudoplastic, non-Newtonian behavior of the viscoelastic polymer. The influence of graphene on the viscoelastic response of the polymer is revealed from the change in the absolute values of the storage (*G’*) and loss (*G”)* moduli, as well as their frequency dependence [33,61]. 

At a high-shear rate, both materials exhibited thinning behavior, which resulted in a decrease of extensional viscosity. However, the incorporation of 1 wt.% graphene increased the melt viscosity of the nanocomposite, though the relative increase gradually lessened at high-shear rate. The presence of graphene has considerably increased the pseudoplasticity at a low shear rate region. At the angular frequency (ω) of 0.0154 rad/s, the complex viscosity increased from 0.13 MPa·s for neat multimodal-HDPE to 0.24 MPa·s for the PE-g-1%. Furthermore, the loss and storage moduli of the neat polymer increased by a value of 92% and 77% with 1 wt.% loading of graphene, respectively. The greater amount of storage and loss moduli of PE-g-1% suggests that the formation of a strong interfacial bonding between the polymer matrix and the high-modulus graphene reduced the loss tangent, the nanocomposite accordingly became more elastic [33,61,62,63]. This is in addition to the thorough dispersion and distribution of the nanofillers, which led to a decrease in the degree of the chain mobility of the polymers, and thus suppressed the shear flow of the polyethylene macromolecular chains [4]. In addition, the crossover modulus point (*G_C_*) and crossover frequency point (*ω_C_*) have decreased from 0.044 MPa·s and 4.5 rad/s for the neat multimodal-HDPE to 0.035 MPa·s and 1.2 rad/s for PE-g-1%, respectively. The shift of *ω_C_* to lower region indicates that the nanocomposite exhibited higher average molecular mass and/or the entangled molecules were induced by the three-dimensional network of graphene platelets within the matrix [33]. However, the G_C_ shifting to lower values after the reinforcement indicates that the polymer exhibited broader molecular weight distribution, which is evident from the increase in the polydispersity index (PDI) by 18.4%, i.e. the larger the PDI, the broader the molecular weight distribution [33]. The shift of *G_c_* upon the addition of the reinforcement possibly arose also from exposing the neat polymer to high temperature, under a combination of high shear and elongation forces, for a prolonged period of time. In Figure 6b, the melt flow rate (MFR) of the extruded multimodal-HDPE decreased gradually from 13.6 g/min to only 6.42 g/min with 1 wt.% graphene loading at 21.6 kg/190 °C, whilst the MFR of the pristine multimodal-HDPE (non-extruded) was only was only 7.5 g/min. The lower the MFR, the higher the molecular weight or the viscosity. This indicates that graphene acted as a thermal barrier and enhanced the thermal stability of the polymer through the strong interface bonding. 

The synergistic effect advantages of graphene are further investigated by thermal expansion and thermogravimetric analyses (TGA). As shown from the TGA thermograms in Figure 6c, the onset degradation temperature of PE-g-1% increased significantly by more 31 °C. The onset temperature at 5% mass loss (T5%) of neat multimodal-HDPE increased from 405 °C to 434.2 °C upon 1 wt.% loading of graphene (see Table 1). The reinforced polymers exhibited a greater melt strength during thermoforming such that the sagging resistance of the nanocomposite has been improved. The large aspect ratio of graphene with a platelet structure likely offered a larger interfacial surface with the polymer matrix which in turn slowed the diffusion of the decomposition products from a continuous network-structured protective layer created in the nanocomposite. It would seem as though graphene acted as an antioxidant and consequently protected the polymer from excessive thermal degradation [64,65,66,67,68,69]. The polymer could therefore be extruded in aggressive conditions, for example with a screw configuration of 37% of dispersive elements at a very low feed rate. This also implies that the amount of thermo-mechanical and thermo-oxidative degradations achieved was sufficient enough to produce an efficient reinforcement. This was achieved through the formation of a strong interfacial adhesion bond between the polymer matrix and graphene platelets, which has accordingly enhanced the thermal stability of the polymer. 

The coefficient of thermal expansion (CTE) of the PE-g-1% was calculated to be 0.55 × 10^−6^ °C^−1^ over the temperature range of 30–103 °C, as shown in Figure 6d-e. The CTE started to become positive after 100 °C confirming that graphene sheets were well bonded with the polymer matrix, and suggests the continuous interconnected network structure formed in the polymer matrix hindered the reorientation of the polymer chains. Mounet et al. [70], Zakharchenko et al. [71], Yoon et al. [72], Bao et al. [73], and others found that graphene has negative thermal expansion at low temperatures. Mounet et al. [70] used a first-principles calculation, and estimated the CTE of graphene remains negative up to 2500 K. Zakharchenko et al. [71] found that the transition from negative to positive CTE occurs at ~900 K. The CTE of a single-layer graphene measured by Yoon et al. [72] via temperature-dependent Raman spectroscopy remained negative in the temperature range of 200–400 K. Therefore, it is not yet clear at what exact point the CTE changes from negative to positive. 

## 4. Conclusions

A high-performance novel graphene-based multimodal polyethylene nanocomposite was produced directly without any particular treatment to the composite constituents. Different characterization techniques such as TEM, SEM, optical microscopes, Raman spectroscopy, Rheology, density measurements, TGA, and CTE were employed in order to demonstrate the novelty of the fabrication method. Microscopic analysis showed that graphene platelets were homogenously dispersed and distributed within the polymer matrix. The electronic and optical microscopies showed that the graphene was dispersed with an average size of less than 450 nm, and distributed with a %area fraction of only 0.0063. The adhesion strength between the graphene sheets and polymer matrix examined by microscopic examination of the cryofractured surfaces of the nanocomposite, mechanical testing and stress-induced Raman band shifts, revealed a strong interfacial bonding attained through thermo-mechanical and thermo-oxidative degradation to a controlled level during the melt extrusion process. The cryofractured surface of the nanocomposite became rough with fibrils almost entirely absent. Deflection of the nanocomposite led the characteristic 2D and G Raman peaks of graphene to shift significantly towards high wavenumbers. This was confirmed further by the mechanical testing where the storage modulus of the polyethylene reinforced with 1 wt.% of graphene increased up to 118% at room temperature.

The thermal performance of the nanocomposite was investigated via rheology testing, TGA and thermal expansion analysis. Loading a polymer with 1 wt.% graphene has resulted in a significant shift of the crossover frequency point and crossover modulus point to the lower regions. The homogenous dispersion and distribution of graphene platelets within the polymer matrix, as well as the strong adhesion bonding, led to the formation of an interconnected 3D network in the polymer which accordingly restricted the movement and expansion of the polymer chains movements. This has resulted in a significant increase in the onset degradation temperature by more 31 °C as a consequence of a thermal barrier or synergetic effects induced by graphene. The nanocomposites exhibited almost zero thermal expansion below 100 °C. The results of this study change the idea of there being difficulty in using melt extrusion for producing well dispersed and disturbed graphene-based hydrophobic polymer nanocomposites. Furthermore, considering there is no need to chemically treat graphene or polymer, or in fact change anything in the existing plants, such a result can significantly attract the industry. 

## Figures and Tables

**Table 1:** TGA data of the neat multimodal-HDPE and its nanocomposite.

Sample	Tonset, (°C)	T5%, (°C)	T30%, (°C)	T50%, (°C)	T80%, (°C)
**Neat multimodal-HDPE**	400	405	406.8	409	419.3
**PE-g-1%**	433.4	435	437	437	437.2

T5%, T30%, T50%, and T80%, are the onset temperatures at 5%, 30%, 50%, and 80% mass loss, respectively.

## Supplementary Materials

The following are available online at http://www.mdpi.com/2079-4991/8/11/947/s1, Figure S1: Type and use of each element used in the present study, Figure S2: The cross-sectional area of a two-flighted screw element, Figure S3: XPS wide range spectrum of graphene powder (CAE=50eV). A C1s (CAE=50eV) narrow scan peak fit is also presented in the inset, showing the components and the degree of oxygen species. They were fitted by the Gaussian-Lorentzian (GL60) functions. Figure S4: TEM images of the as-received graphene powders at different magnifications.
