# Supplementary material for: Advancing the Use of High-Performance Graphene-Based Multimodal Polymer Nanocomposite at Scale
Source: Nanomaterials (Basel). 2018 Nov 17;8(11):947. doi: 10.3390/nano8110947 (PMC6266415; doi:10.3390/nano8110947)
Supplement: Supplementary file 1 [file nanomaterials-08-00947-s001.pdf]

Electronic Supplementary Information (ESI)

Advancing the Use of High-Performance Graphene-Based Multimodal Polymer Nanocomposite at Scale

Ibrahim A. Ahmad [1], Krzysztof K. K. Koziol [2], Suleyman Deveci [3], Hyun-Kyung Kim [1,4,*] and Ramachandran Vasant Kumar [1,*]

1 Department of Materials Science and Metallurgy, University of Cambridge, 27 Charles Babbage Rd, Cambridge CB3 0FS, UK; iaiaa2@cam.ac.uk
2 Enhanced Composites and Structures Centre, School of Aerospace, Transport and Manufacturing, Cranfield University, Cranfield MK43 0AL, UK; k.koziol@cranfield.ac.uk
3 Innovation Centre, Borouge Pte. Ltd., PO Box 6951, Abu Dhabi, UAE; suleyman.deveci@borouge.com
4 Gwangju Bio/Energy R&D Center, Korea Institute of Energy Research (KIER), 270-25 Samso-ro, Buk-gu, Gwangju 61003, Korea
* Correspondence: hkk28@cam.ac.uk (H.-K.K.); rvk10@cam.ac.uk (R.V.K.); +44 (0)1223 334327

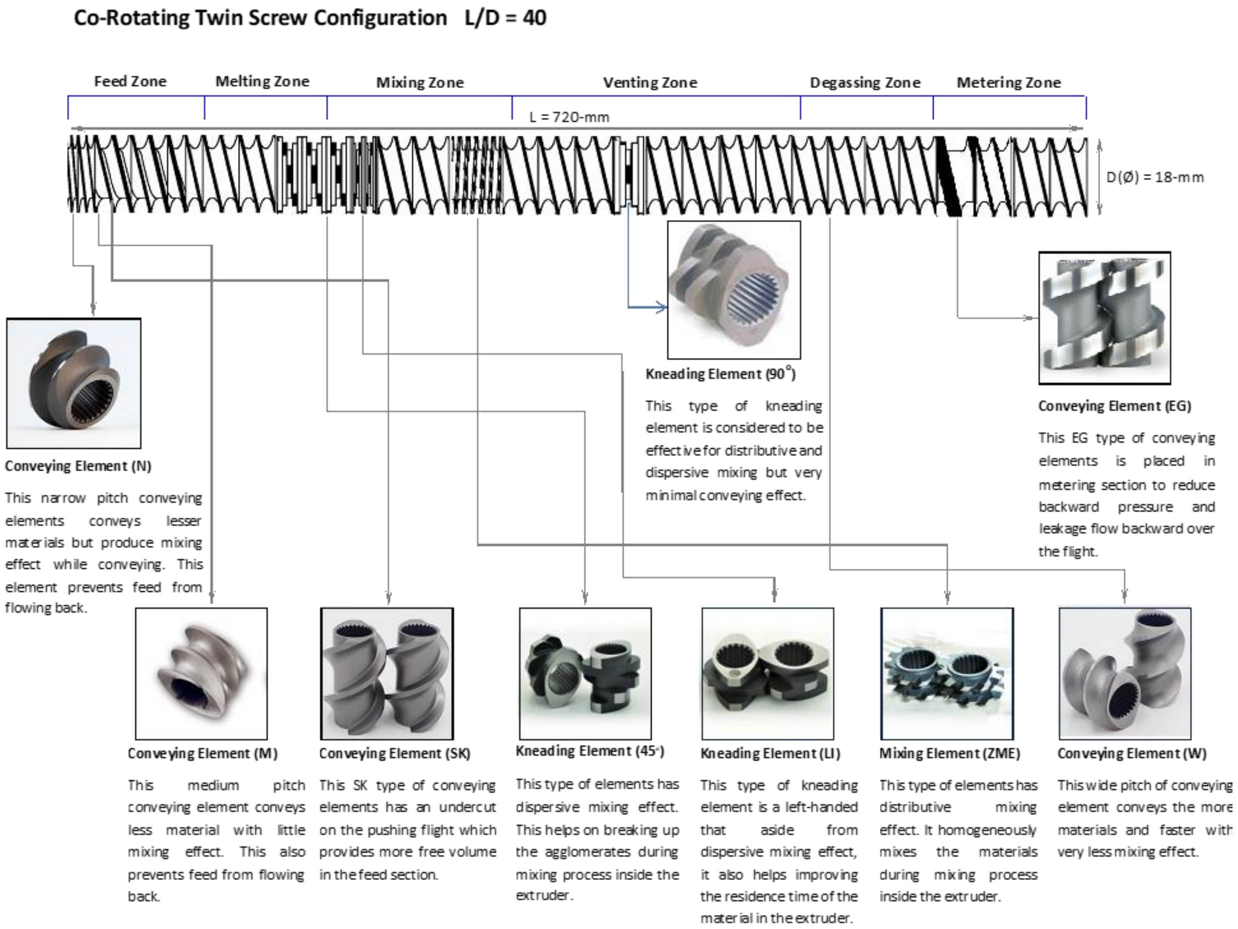

Figure S1. Type and use of each element used in the present study.

As the screws are rotating in the same direction, each screw picks up the material and drags it towards the intermeshing region shown in Figure S2 [1–6]. However, because the two screws are closely intermeshed, the materials have little opportunity to pass through the intermeshing regions, and subsequently move in a figure-of-eight pattern, as well as in an axial direction [3–8]. Nevertheless, these materials will be obstructed by the width of the adjacent screw and form a circulatory flow if they are close to the flight flank [9]. Therefore, transport of the materials is restricted proportionally by increasing the width of the kneading block elements [3]. Dispersive mixing, or exfoliation of nano-fillers is carried out by an aggressive function of the kneading blocks, occurring in the clearances between the flight tips of the element and the intermeshing zones [9].

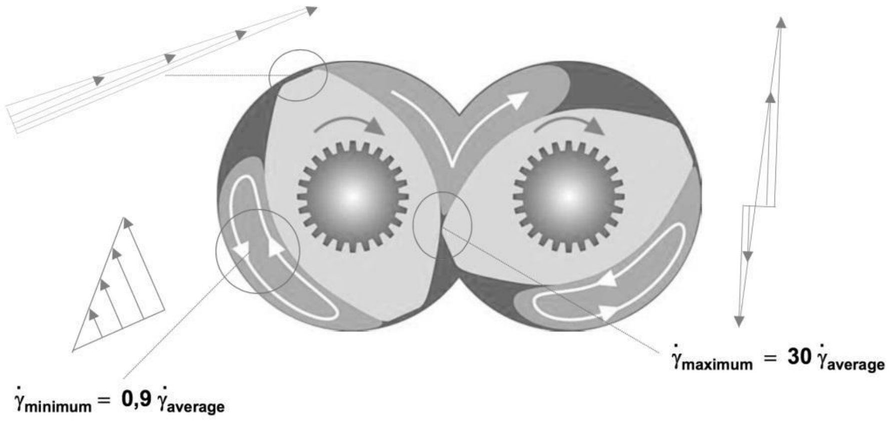

Figure S2. The cross-sectional area of a two-flighted screw element. Shear rate $\dot{\gamma}$ is the circumferential to speed/channel depth. Reproduced from [1].

In these regions, the polymer melt experiences combined forces of shear and elongation, as shown in Figure S3 [8]. However, effective distributive mixing is achieved by circulating flow in the melt pools at a low shear rate, and separating in the intermeshing zone [7]. N. Kim and coworkers analyzed the non-Newtonian flow using a Carreau-Yasuda model, observing that the temperature had increased significantly where the deformation rate was high, owing to the heat generated by the viscous dissipation [7]. The staggering angle in the kneading blocks also affected the efficiency of the distributive mixing, where it produced a backflow between the adjacent discs at a higher angle. The staggering angle was 45 °for most of the kneading elements used in this study. Moreover, compressing the fluid by advancing the discs has increased the residence time in this region [7]. Mixing the molten is dispersive in nature because of the high

viscosity and shear stress induced by the polymer melt [1]. Also, the heat required to melt the polymer is transferred by conduction, heated barrel, and convection [10]. However, the mechanical energy in a co-rotating twin screw extruder is mainly dissipated into heat by friction energy dissipation, plastic energy dissipation, and viscous energy dissipation [10]. The melting zone must, therefore, contain kneading elements followed by backflow elements for efficient melting [6].

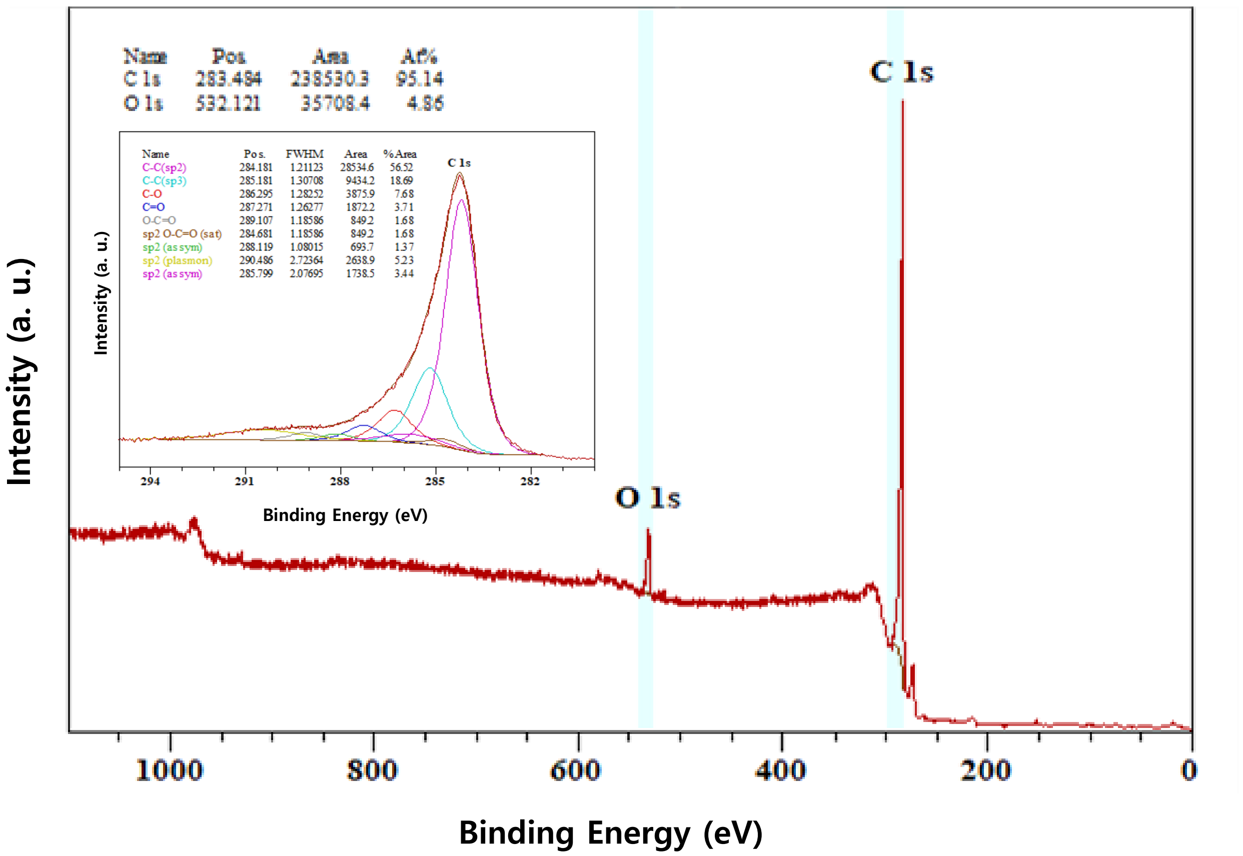

Figure S3. X-ray photoelectron spectroscopy (XPS) wide range spectrum of graphene powder (CAE=50eV). A C1s (CAE=50eV) narrow scan peak fit is also presented in the inset, showing the components and the degree of oxygen species. They were fitted by the Gaussian-Lorentzian (GL60) functions.

Using a VG Scientific ESCALAB 200-D spectrometer equipped with Al Kα (non-monochromated) at 12keV and 20mA, X-ray photoelectron spectroscopy (XPS) was undertaken to determine the C/O ratio of the graphene powder, obtained from the supplier detailed in the Materials section. Figure S4 shows elemental sampling of the as-received graphene over a wide

range spectrum, between 0 and 1100 eV, and the major peaks of C1s and O1s appeared at 283–294 eV and 534-535 eV, respectively. They are fitted according to ref. 12. The recorded C/O ratio was 19.6.

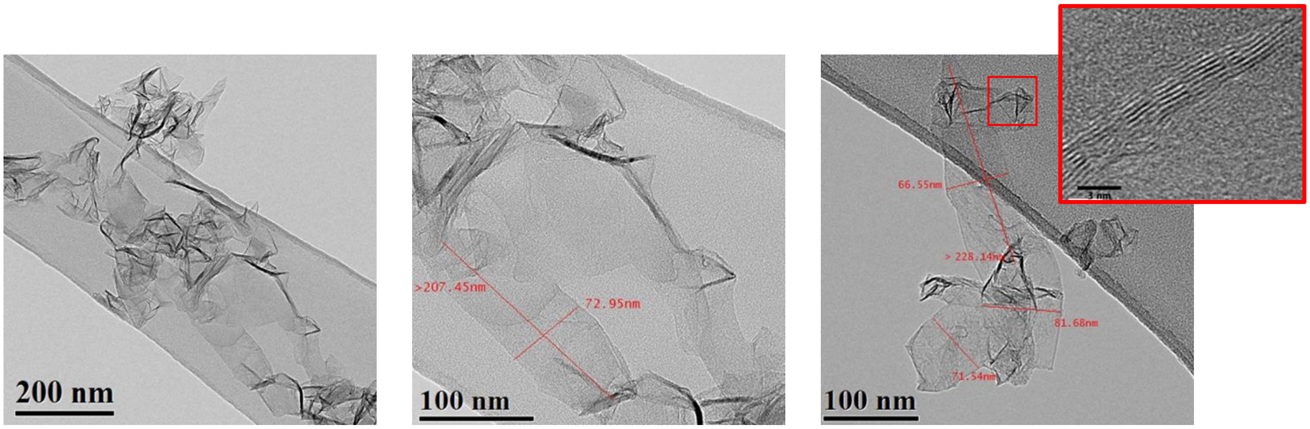

Figure S4. Transmission electron microscope (TEM) images of the as-received graphene powders at different magnifications.

Analysis was performed using a JEOL JEM2100FCs Field Emission transmission electron microscope (TEM), with a CEOS aberration corrected illumination system. This was performed at the standard operating voltage of 200kV and in normal Bright Field illumination mode, with a standard 3 mm lacey-carbon support copper grid (see Figure S4). This mode allows limitation of the localized current density of the high brightness beam, as beam-induced damage is expected to be a potential issue. Of the observed less-disturbed platelets, the average lateral dimensions were generally in the order of 220 nm, although there is a tendency for larger values along one dimension, often exceeding 200 nm. High magnification shows that the graphene is composed of about 5-7 layers. The clusters still showed a predominance of edge-wrapping and overlapping, particularly at the film edges.

Reference

1. Schonfeld, S. Compounding of Filled Polymers with the Co-rotating Twin Screw Extruder ZSK; Coperion: Stuttgart, Germany, 2013.
2. Jansen L.P.; Moscicki, L. *Thermoplastic Starch*, 1st ed.; WILEY-VCH: Darmstadt, Germany, 2009.

3. Rauwendaal, C. *Polymer Extrusion*, 5th ed.; Hanser Gardner Publications: Munich, Germany, 2014.
4. Sakai, T. Screw extrusion technology—Past, present and future. *Polimery* **2013**, *58*, 847–857.
5. Chiruvella, R.V.; Jaluria, Y.; Karwe, M.V.; Sernas, V. Transport in a twin-screw extruder for the processing of polymers. *Polym. Eng. Sci.* **1996,** *36*, 1531–1540.
6. Montiel, R.; Patiño-Herrera, R.; Gonzalez-Calderón, J.A.; Pérez, E. Novel twin screw co-extrusion-electrospinning apparatus. *Am. J. Biomed. Eng*. **2016**, *6*, 19–24.
7. Kim, N.; Kim, H.; Lee, J. Numerical analysis of internal flow and mixing performance in polymer extruder II: twin screw element. *Korea-Aust. Rheol. J.* **2006**, *18*, 153–160.
8. Zhang, H.; Huang, J.; Yang, L.; Chen, R.; Zou, W.; Lin, X.; Qu, J. Preparation, characterization and properties of PLA/TiO2 nanocomposites based on a novel vane extruder. *RSC Adv.* **2015,** *5*, 4639–4647.
9. Jeon, I.Y.; Baek, J.B. Nanocomposites derived from polymers and inorganic nanoparticles. *Materials* **2010**, *3*, 3654–3674.
10. Wilkinson, A.N.; Ryan, A.J. *Polymer Processing and Structure Development*, 1st ed.; Kluwer Academic Publishers group: Dordrecht, Germany, 1999.
